# miRNA-146a Mimic Inhibits NOX4/P38 Signalling to Ameliorate Mouse Myocardial Ischaemia Reperfusion (I/R) Injury

**DOI:** 10.1155/2021/6366254

**Published:** 2021-07-27

**Authors:** Lili Xiao, Yulei Gu, Gaofei Ren, Linlin Chen, Liming Liu, Xiaofang Wang, Lu Gao

**Affiliations:** ^1^Department of Cardiology, The First Affiliated Hospital of Zhengzhou University, Zhengzhou, Henan Province, China; ^2^Emergency Intensive Care Unit, The First Affiliated Hospital of Zhengzhou University, Zhengzhou, Henan Province, China; ^3^Endocrinology Department, The First Affiliated Hospital of Zhengzhou University, Zhengzhou, Henan Province, China

## Abstract

Evidence suggests that miR-146a is implicated in the pathogenesis of cardiovascular diseases; however, the role of miR-146a in myocardial ischaemia reperfusion (I/R) injury is unclear. The aim of this study was to explore the functional role of miR-146a in myocardial ischaemia reperfusion injury and the underlying mechanism. C57BL/6J mice were subjected to 45 min of ischaemia and 1 week of reperfusion to establish a myocardial I/R injury model. A miR-146a mimic (0.5 mg/kg) was administered intravenously at the beginning of the ischaemia process. Neonatal rat cardiomyocytes were also subjected to hypoxia/reperfusion (H/R). Cells were treated with the miR-146a mimic or antagonist. As a result, the miR-146a mimic attenuated H/R-induced cardiomyocyte injury, as evidenced by increased cell viability and reduced lactate dehydrogenase (LDH) levels. In addition, the miR-146a mimic inhibited oxidative stress in cells suffering from H/R injury. Moreover, the miR-146a antagonist exerted adverse effects in vitro. In mice with myocardial I/R injury, the miR-146a mimic preserved cardiac function and reduced the infarction area and fibrosis. Moreover, the miR-146a mimic decreased the inflammatory response and reactive oxygen species (ROS) accumulation in mouse hearts. Mechanistically, we found that miR-146a directly regulated the transcription of NOX4, which subsequently affected P38 signalling in cardiomyocytes. When we knocked down NOX4, the effects of the miR-146a antagonist in worsening the cell condition were counteracted in in vitro experiments. Taken together, the results suggest that miR-146a protects against myocardial ischaemia reperfusion injury by inhibiting NOX4 signalling. The miR-146a mimic may become a potential therapeutic approach for patients with myocardial ischaemia reperfusion.

## 1. Introduction

In clinical emergencies worldwide, the incidence of acute myocardial infarction (AMI) is particularly alarming, ranking first in the world. The most effective way to reduce early AMI injury and infarction is timely coronary revascularization via thrombolytic therapy or percutaneous coronary stent implantation (PCI) [1]. Although myocardial reperfusion is a recognized method of reducing injury, myocardial reperfusion itself can trigger a variety of pathological reactions, leading to myocardial cell death [2]. The mechanisms underlying myocardial ischemia reperfusion injury (IRI) include oxidative stress, Ca^2+^ overload, and mitochondrial permeability transition pore (mPTP) opening [3]. It has been proven difficult to translate cardioprotective measures into clinical practice. Although ischaemic conditioning strategies are promising, their transformation effect is weak [4]. Therefore, it is very important to explore the mechanism underlying IRI and identify new cardioprotective measures.

The reactive oxygen species (ROS) burst during myocardial reperfusion is the primary cause of myocardial cell death, leading to the opening of the mPTP, which plays a critical role in reperfusion damage [5]. Oxidative stress can affect the expression level of miRNAs, and miRNAs can regulate oxidative stress by regulating the expression of redox sensor proteins [6]. Although miRNAs do not encode proteins, they play a key role in the regulation of gene expression at the posttranscriptional level [6]. miR-146a has been widely recognized as a critical regulatory element in the cardiovascular system. miR-146a was reported to regulate mitochondrial function in a myocardial infarction mouse model [7, 8]. miR-146a also protected against myocardial fibrosis in a rat constrictive pericarditis model [8]. miR-146a protected against doxorubicin-induced cardiotoxicity by modulating autophagy [9].

All this evidence suggests a protective effect of miRNA-146a in cardiovascular disease. In this study, we aimed to explore whether a miRNA-146a mimic could protect against myocardial reperfusion injury as well as the underlying mechanism.

## 2. Methods

### 2.1. Animals

C57BL/6J male mice (8 weeks old, 24-25 g) were purchased from the Chinese Academy of Medical Sciences (Beijing, China) and raised in the SPF Laboratory Animal Center of Zhengzhou University. The mice were divided into 4 groups (*n* = 12 for each group): vehicle-sham, miR-146a mimic-sham, vehicle-I/R, and miR-146a mimic-I/R. The mice were administered with intracoronary injections of 0.5 mg/kg miR-146a mimics (mmu-miR-146a, Fermentas GmbH, Germany) formulated [10] as lipoplexes with DMAPAP/DOPE cationic liposomes at the beginning of ischaemia [11]. The control mice were injected with lipoplexes containing irrelevant miRNA mimics. All the animal experiments were approved by the Institutional Animal Care and Use Committee of Zhengzhou University (Zhengzhou, China).

### 2.2. Animal Model

The model of I/R injury was established by 45 min of ischaemia and 1 week of reperfusion. Left coronary artery ligation surgery (LAD) was conducted according to previously published protocols [12, 13]. In short, after anaesthetization, the mouse chest was opened on the left side between the third and fourth intercostal spaces. After opening the pericardium, the proximal descending branch of the LAD was ligated with 7~0 silk thread. After 45 min of ischaemia, the ligation was released, and the heart was reperfused for 1 week. The LAD was not ligated in the sham group.

### 2.3. Echocardiographic Evaluation

Transthoracic echocardiography was performed as previously described [13, 14]. Isoflurane (1.5%) was used to anaesthetize the mice, and echocardiography was performed with a 10 MHz linear array ultrasound transducer to obtain M-mode echocardiography data. The left ventricle (LV) end-diastolic dimension (LVEDd) and LV end-systolic dimension (LVESd) were obtained, and the LV ejection fraction (LVEF) and LV fractional shortening (LVFS) values were calculated. A total of 10 mice from each group were subjected to transthoracic echocardiography.

### 2.4. Triphenyltetrazolium Chloride, PSR Staining, and Immunofluorescence Staining

Triphenyltetrazolium chloride (TTC, 1%, Sigma, USA) staining was used to evaluate the MI area and morphological changes in the heart. The hearts were cut into 1-2 mm thick myocardial sections along the long axis of the heart. For the infarct area calculation, Image-Pro Plus 6.0 was used to analyse 5 sections from each heart and 6 hearts from each group. PSR staining was used to show the collagen volume. For the fibrosis area calculation, Image-Pro Plus 6.0 was used to analyse 6 sections from each heart and 6 hearts from each group. Macrophages were subjected to immunofluorescence staining to assess CD68 expression. After dehydration, antigen repair was conducted at a high temperature and pressure, and the sections were sealed with 8% goat serum. The heart sections were incubated with an anti-CD68 antibody (Abcam, 1 : 100 dilution). A secondary antibody, goat anti-rabbit IRdye 800CW (LI-COR), was used. The nucleus was stained with DAPI. We counted the number of CD68-positive cells in each group (10 fields for each heart). Dihydroethidium (DHE) staining (Sigma-Aldrich) was performed to identify intracellular ROS production. Mean DHE fluorescence was calculated by subtracting the integrated density of the background signal from the integrated density of the fluorescent staining from 10 fields/heart and 5 hearts/group, and the results were normalized to the control.

### 2.5. Cardiomyocyte Isolation and Culture

Neonatal rat cardiomyocyte (NRCM) culture was performed as previously described [13, 14]. Briefly, the hearts of Sprague-Dawley rats (1-3 days old) were quickly removed, and ventricles were preserved and digested with 0.125% trypsin-EDTA (Gibco) 4 times for 15 min each time. Digestion was stopped with DMEM-F12 supplemented with 15% foetal bovine serum (FBS, Gibco, USA). After 5 digestion reactions, the cells were collected and incubated in a 100 mm dish with DMEM-F12 supplemented with 15% FBS. After 90 min, the cell culture medium was collected, and NRCMs in the upper layer of the cell medium were removed and seeded onto a 6-well plate to exclude the noncardiac myocytes that adhered to the bottom of the 100 mm dish. NRCMs were identified by *α*-actin staining.

For the H/R model, cardiomyocyte culture medium was converted into H/R buffer (4 mM HEPES, 12 mM KCl, 117 mM NaCl, 0.49 mM MgCl_2_, 0.9 mM CaCl_2_, 20 mM sodium lactate, and 5.6 mM 2-deoxyglucose, pH 6.2) and placed in a hypoxic chamber (95% N_2_/5% CO_2_, Billups-Rothenberg) at 37°C for 30 min [15]. The chamber was closed, and a normal oxygen supply was added for an additional 4 hours, followed by reperfusion with DMEM supplemented with 10% FBS. NRCMs were transfected with miR-146a mimics, miR-146a inhibitor (Exiqon, Denmark), or their corresponding negative controls at a final concentration of 50 nM for 24 h using Lipofectamine 2000 before the H/R model was established in accordance with the manufacturer's instructions. NRCMs were transfected with NOX4 siRNA (RiboBio, China) to knock down NOX4. Cell viability was detected with a CCK8 kit (Beyotime Biotechnology, Shanghai, China) with a microplate reader measuring the intensity of the light absorption at 450 nm wavelength.

### 2.6. ELISA Detection of Inflammatory Cytokines

The tumour necrosis factor *α* (TNF*α*) and interleukin- (IL-) 1 levels in mouse hearts and cardiomyocytes were detected with ELISA purchased from BioLegend (430901, 432604). An ELISA instrument (Synergy HT, BioTek, United States) was used to measure the absorbance.

### 2.7. Oxidative Stress Assessment

The activities of manganese superoxide dismutase, superoxide dismutase 2 (MnSOD), nicotinamide adenine dinucleotide phosphate (NADPH) oxidase, and malondialdehyde (MDA) in heart tissues and cardiomyocytes were detected by using corresponding kits purchased from Beyotime (Shanghai, China) according to the manufacturer's instructions [13]. The level of ROS was measured according to a previous study [16] using 2′,7′-dichlorofluorescin diacetate (DCFH-DA) and an ELISA plate reader (Synergy HT, BioTek, Vermont, USA).

### 2.8. Cardiac Troponin I (cTNI) and Lactate Dehydrogenase (LDH) Release

After 2 hours of reperfusion, venous blood was collected and centrifuged at 4000 r/min at 4°C for 10 min, and serum was collected to detect the concentration of cTNI. All the procedures were performed in accordance with the instructions of the kit (Nanjing Jiancheng Biological Company) with an ELISA plate reader (Synergy HT, BioTek, Vermont, USA). The levels of LDH released were detected in the cell supernatant using an LDH release assay kit (Nanjing Jian) according to the manufacturer's instructions.

### 2.9. Western Blotting and qPCR

Total protein was isolated from heart tissues and NRCMs and then subjected to SDS-PAGE (50 *μ*g per sample). After transfer onto Immobilon membranes (Millipore, Billerica, MA, USA), proteins were incubated overnight at 4°C with primary antibodies against NOX2 (ab129068) and NOX4 (ab133303), which were purchased from Abcam (1 : 1000 dilution), and phosphorylated and total P38 (4511P/9212P) and GAPDH (2118), which were purchased from Cell Signaling Technology (1 : 1000 dilution). The blots were developed with enhanced chemiluminescence (ECL) reagents (Bio-Rad, Hercules, CA, USA), and images were captured by using a ChemiDoc MP Imaging System (Bio-Rad). GAPDH served as an internal reference protein.

Total RNA (2 *μ*g per sample) from frozen mouse heart tissue and cardiomyocytes was reverse transcribed into cDNA using oligonucleotide (DT) primers and a transcript first-strand cDNA synthesis kit (Roche). Then, a LightCycler 480 instrument (software version 1.5, Roche) and SYBR Green PCR Master Mix (Roche) were used to perform RT-PCR. All the genes were normalized using GAPDH.

### 2.10. Luciferase Assay

The full-length 3′UTR of NOX4 was amplified by PCR (forward: 5′-CGCCTCCCGGGTTTGCACCACTCTCCTGCCTCAGCCTCCTG-3′; reverse: 5′-ATCATTTTTATTGTCTCAAGAAGAACTTAATAGCAATTAG-3′). The NOX4 3′-UTR sequences were inserted into the pmirGLO vector (Guangzhou Boxin Biotechnology Co., Ltd.). The empty particle vector was used as control. NRCMs were cotransfected with the reporter plasmid, miR-146a mimic, inhibitor, or negative controls for 24 h. A GloMax® 20/20 Luminometer (Promega) was used to detect luciferase.

### 2.11. Statistical Analysis

All the data are expressed as the mean ± SD. Differences among groups were analysed by two-way analysis of variance followed by Tukey's post hoc test. Comparisons between two groups were analysed by unpaired Student's *t*-test. *P* values less than 0.05 indicated statistical significance.

## 3. Results

### 3.1. miR-146a Protects NRCMs from H/R-Induced Inflammation

**Figure 1:**
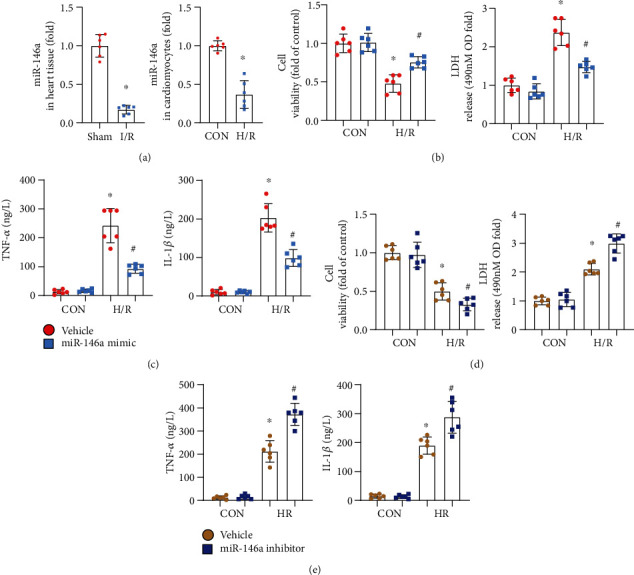
miR-146a protects NRCMs from H/R-induced inflammation. (a) The mRNA level of miR-146a in heart tissue after I/R injury (*n* = 6) and in cardiomyocytes after H/R injury (*n* = 6). (b, c) NRCMs were transfected with the miR-146a mimic and subjected to H/R injury. (b) Cell viability detected by the CKK8 assay and LDH release in each group (*n* = 6). (c) TNF*α* and IL-1*β* production in cells detected by the ELISA (*n* = 6). ^∗^*P* < 0.05*vs.* vehicle-CON; ^#^*P* < 0.05*vs.* vehicle-H/R. (d, e) NRCMs were transfected with the miR-146a inhibitor and subjected to H/R injury. (d) Cell viability detected by the CKK8 assay and LDH release in each group (*n* = 6). (e) TNF*α* and IL-1*β* production in cells detected by ELISA (*n* = 6). ^∗^*P* < 0.05*vs.* vehicle-CON; ^#^*P* < 0.05*vs.* vehicle-H/R. Cell experiments were repeated for 3 times. Unpaired Student's *t*-test was used in (a); two-way analysis of variance was used in (b)–(e).

We first evaluated the expression of miR-146a in heart tissues and cardiomyocytes after I/R injury. miR-146a expression was reduced in heart tissue after I/R injury and decreased in cardiomyocytes after H/R injury (Figure 1(a)). We then evaluated the effect of the miR-146a mimic on H/R injury in cardiomyocytes. As shown in Figure 1(b), cell viability was reduced in H/R cardiomyocytes, while the miR-146a mimic increased cell viability when cells were treated with the miR-146a mimic before exposure to H/R injury. In addition, cells treated with the miR-146a mimic showed less LDH release and reduced TNF*α* and IL-1 levels (Figure 1(c)). These results suggest that the miR-146a mimic could attenuate H/R-induced cardiomyocyte injury and inflammation. Next, we explored the role of the miR-146a inhibitor. The miR-146a inhibitor exacerbated cardiomyocyte H/R injury, as assessed by lower cell viability and higher LDH release (Figure 1(d)) as well as higher TNF*α* and IL-1 levels (Figure 1(e)) than the vehicle-treated group. These data suggest that miR-146a protects against cardiomyocyte H/R injury.

### 3.2. miR-146a Protects NRCMs from H/R-Induced Oxidative Stress

**Figure 2:**
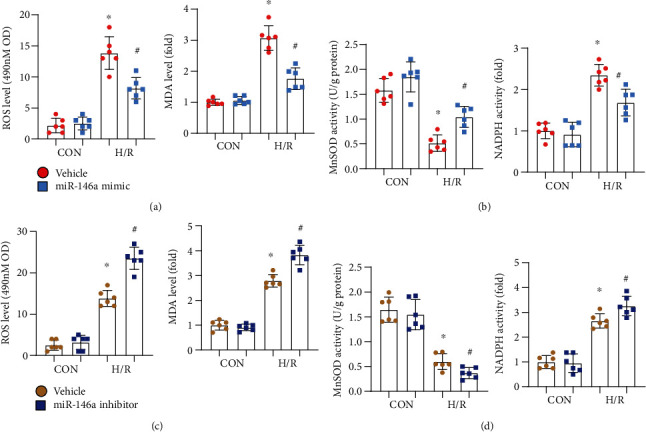
miR-146a protects NRCMs from H/R-induced oxidative stress. (a, b) NRCMs were transfected with the miR-146a mimic and subjected to H/R injury. (a) ROS level detected by the 2′,7′-dichlorofluorescin diacetate probe and MD level in each group (*n* = 6). (b) MnSOD and NADPH oxidase activity in cells (*n* = 6). ^∗^*P* < 0.05*vs.* vehicle-CON; ^#^*P* < 0.05*vs.* vehicle-H/R. (c, d) NRCMs were transfected with the miR-146a inhibitor and subjected to H/R injury. (c) ROS level detected by the 2′,7′-dichlorofluorescin diacetate probe and MD level in each group (*n* = 6). (d) MnSOD and NADPH oxidase activity in cells (*n* = 6). ^∗^*P* < 0.05*vs.* vehicle-CON; ^#^*P* < 0.05*vs.* vehicle-H/R. Cell experiments were repeated for 3 times. Two-way analysis of variance followed by Tukey's post hoc test was used.

Oxidative stress is the main cause of cell death in I/R injury. We then detected whether miR-146a influences the redox balance in cardiomyocyte H/R injury. ROS levels were reduced in cells treated with the miR-146a mimic (Figure 2(a)). The levels of MDA, the intermediate metabolite of lipid peroxidation, were also lower in cells treated with the miR-146a mimic (Figure 2(a)). The antioxidant activity and MnSOD activity were elevated, but NADPH oxidase activity was reduced in cells treated with the miR-146a mimic (Figure 2(b)). In cells treated with the miR-146a inhibitor, the ROS level was elevated (Figure 2(c)) after H/R injury. In addition, MDA levels and NADPH oxidase activity were also increased, while MnSOD activity was decreased in cells treated with the miR-146a inhibitor (Figure 2(d)). Thus, miR-146a protects against cardiomyocyte H/R injury by inhibiting oxidative stress.

### 3.3. miR-146a Mimic Attenuates Mouse I/R Injury

**Figure 3:**
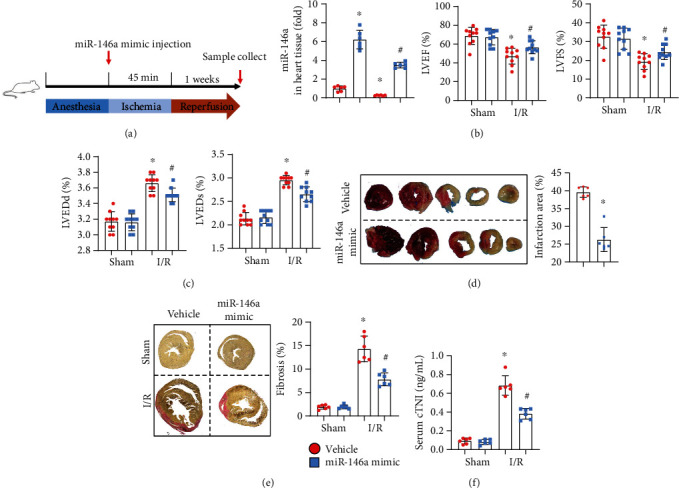
miR-146a mimic attenuates mouse I/R injury. (a) The procedure of mouse experiments. (b) The mRNA level of miR-146a in mouse heart 1 week after injection (*n* = 6). (c) Echocardiography results of LVEF, LVFS, LVEDd, and LVESd in each group (*n* = 10). (d) TTC staining image and quantitative results (*n* = 6). (e) PSR staining image and quantitative results (*n* = 6). (f) cTNI concentration in mouse serum 2 h after I/R injury. ^∗^*P* < 0.05*vs.* vehicle-sham; ^#^*P* < 0.05*vs.* vehicle-I/R. Two-way analysis of variance was used in (b), (c), (e), and (f). Unpaired Student's *t*-test was used in (d).

Mice were subjected to I/R injury to confirm the protective effect of miR-146a in vivo. The miR-146a mimic was injected into mice at the beginning of ischaemia. One week postreperfusion, mice were subjected to echocardiogram to detect cardiac function (Figure 3(a)). miR-146a expression was upregulated in mouse hearts in both the sham group and the I/R group (Figure 3(b)). LVEF and LVFS were reduced in the I/R group, while LVEDd and LVEDs were elevated in the I/R group compared to the sham group. Mice in the miR-146a mimic group showed improved cardiac function, as assessed by increased LVEF and LVFS and reduced LVEDd and LVESd (Figure 3(c)). The infarction area was also detected by TTC staining. Mice treated with the miR-146a mimic showed diminished infarction size (Figure 3(d)). The fibrotic area was also diminished in mice treated with the miR-146a mimic, as assessed by PSR staining (Figure 3(e)). After 2 hours of reperfusion, the serum cTNI level was reduced in miR-146a mimic-treated mice compared with vehicle-treated mice (Figure 3(f)).

### 3.4. miR-146a Mimic Inhibits Inflammation and Oxidative Stress in I/R Mice

**Figure 4:**
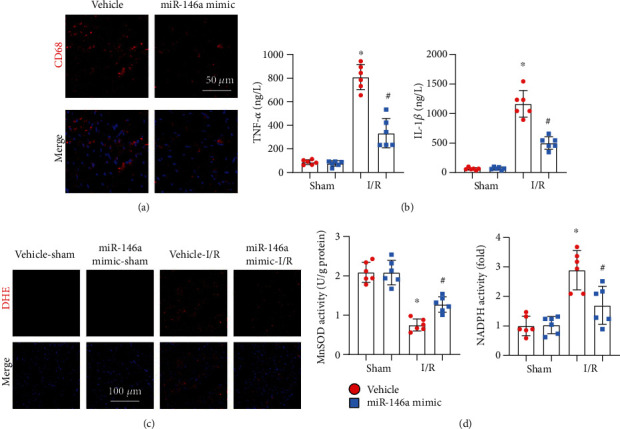
miR-146a mimic inhibits inflammation and oxidative stress in I/R mice. (a) CD68 staining in heart tissue image (*n* = 6). (b) TNF*α* and IL-1*β* production in heart tissue in mice with miR-146a mimic treatment (*n* = 6). (c) ROS level detected by DHE staining in heart tissue (*n* = 6). (d) MnSOD and NADPH oxidase activity in heart tissue (*n* = 6). ^∗^*P* < 0.05*vs.* vehicle-sham; ^#^*P* < 0.05*vs.* vehicle-I/R. Two-way analysis of variance was used in (b) and (d).

Inflammation and oxidative stress were also assessed in vivo. The miR-146a mimic decreased the inflammatory response, as evidenced by reduced CD68-labelled macrophage infiltration and reduced levels of TNF*α* and IL-1 (Figures 4(a) and 4(b)). The miR-146a mimic also decreased the ROS level in I/R-injured hearts, as assessed by DHE staining (Figure 4(c)). The miR-146a mimic increased MnSOD activity and diminished NADPH oxidase activity in mouse hearts after I/R injury (Figure 4(d)).

### 3.5. miR-146a Affects NOX4-P38 Signalling

**Figure 5:**
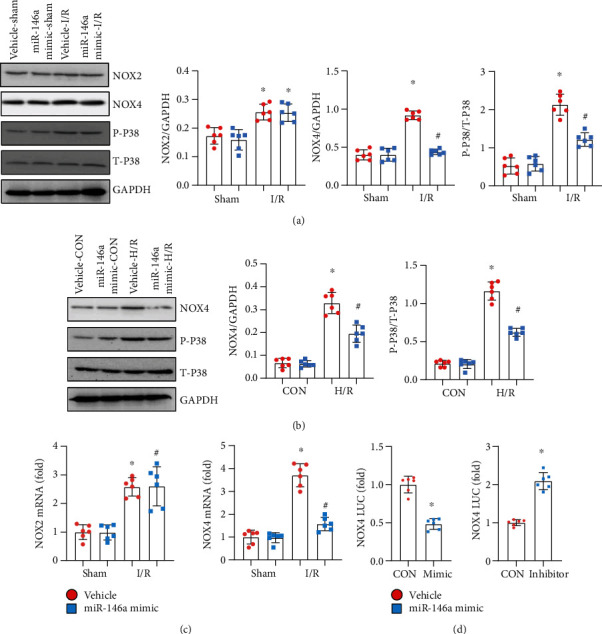
miR-146a affects NOX4-P38 signalling. (a) Protein level of NOX2, NOX4, phosphorylated- (P-) P38, and total- (T-) P38 in heart tissue in mice with miR-146a mimic treatment (*n* = 6). (b) Protein level of NOX4, P-P38, and T-P38 in NRCMs transfected with the miR-146a mimic (*n* = 6). (c) mRNA level of NOX2 and NOX4 in NRCMs transfected with the miR-146a mimic (*n* = 6). (d) Luciferase assay in NRCMs transfected with the miR-146a mimic and miR-146a inhibitor (*n* = 6). Cell experiments were repeated for 3 times. ^∗^*P* < 0.05*vs.* vehicle-sham/CON; ^#^*P* < 0.05*vs.* vehicle-I/R or vehicle-H/R. Two-way analysis of variance was used in (a)–(c). Unpaired Student's *t*-test was used in (d).

To explore the mechanism by which miR-146a inhibits oxidative stress, we detected the levels of proteins associated with ROS production and found that NOX4 but not NOX2, the main subtype of NADPH oxidase expressed in hearts, was inhibited by miR-146a (Figure 5(a)). We also observed that miR-146a reduced the NOX4 level in cardiomyocytes suffering from H/R injury (Figure 5(b)). We then detected whether miR-146a affects NOX4 at the transcriptional level. qPCR results revealed that miR-146a reduced the transcription of NOX4 but not NOX2 in cardiomyocytes (Figure 5(c)). We then hypothesized that miR-146a may directly regulate the promoter region of NOX4 to inhibit its transcription. To test our hypothesis, we performed the luciferase assay. As shown in Figure 5(d), we observed a significant decrease in NOX4-Luc transcriptional activity in miR-146a-treated cells. The use of the miR-146a inhibitor increased NOX4-Luc transcriptional activity.

### 3.6. NOX4 Silencing Abolishes the Effect of the miR-146a Inhibitor

**Figure 6:**
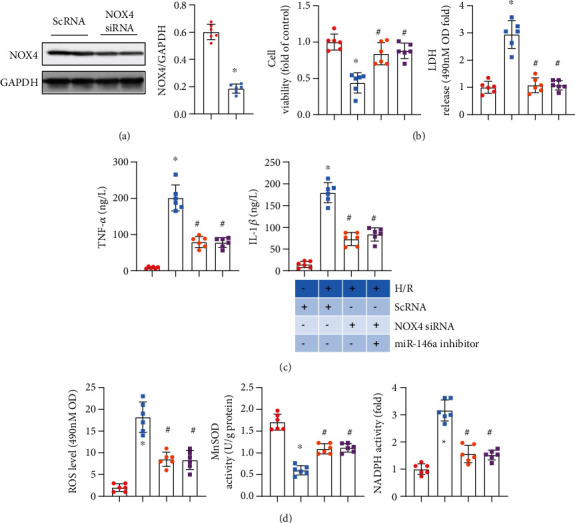
NOX4 silencing abolishes the effect of the miR-146a inhibitor. (a) NRCMs were transfected with NOX4 siRNA (*n* = 6). The protein level of NOX4 after 48 h of transfection (*n* = 6). ^∗^*P* < 0.05*vs.* ScRNA. (b–d) NRCMs were transfected with the miR-146a inhibitor and/or NOX4 siRNA and subjected to H/R injury. (b) Cell viability detected by the CKK8 assay and LDH release in each group (*n* = 6). (c) TNF*α* and IL-1*β* production in cells detected by ELISA (*n* = 6). (d) ROS level and MnSOD and NADPH oxidase activity in cells (*n* = 6). ^∗^*P* < 0.05*vs.* ScRNA-CON; ^#^*P* < 0.05*vs.* ScRNA-H/R. Cell experiments were repeated for 3 times. Unpaired Student's *t*-test was used in (a); one-way analysis of variance was used in (b)–(d).

To confirm the targeting role of miR-146a in NOX4, we performed reverse experiments in vitro. Cells were treated with NOX4 siRNA to knock down NOX4 and with the miR-146a inhibitor. In cells treated with NOX4 siRNA, the expression of NOX4 was decreased significantly (Figure 6(a)). NOX4 knockdown relieved cardiomyocyte H/R injury, as assessed by increased cell viability, reduced LDH release, and diminished TNF*α* and IL-1 levels (Figures 6(b) and 6(c)). NOX4 knockdown dramatically reduced ROS levels and increased MnSOD activity (Figure 6(d)). However, cells treated with both the miR-146a inhibitor and NOX4 siRNA showed no deteriorating H/R injury, as determined by the same extent of increased cell viability and reduced LDH release, inflammation, and oxidative stress compared with cells treated merely with NOX4 siRNA (Figures 6(b)–6(d)). These data indicate that miR-146a protects against myocardial I/R injury by targeting NOX4 expression.

## 4. Discussion

Myocardial ischaemia-reperfusion injury is organ damage caused by reopening of the blood supply after acute myocardial infarction [4]. During myocardial ischaemia, limited oxygen supply is associated with acidosis, energy consumption, and ion homeostasis changes, leading to cell death and cardiac dysfunction [2]. During reperfusion, in the presence of oxygen, ROS are produced in the myocardium [17]. Several miRNAs have been reported to regulate the redox system [6]. Among these, miR-146a is reported as a negative regulator of many cardiovascular diseases, such as myocardial infarction, doxorubicin-induced cardiotoxicity, and cardiac fibrosis in a constrictive pericarditis model [7–9]. Studies have described that miR-146a negatively regulates ROS generation in H9c2 cardiomyocytes [18]. Here, in this study, we demonstrated that the miR-146a mimic could ameliorate cardiomyocyte H/R injury, while the miR-146a inhibitor aggravated cardiomyocyte H/R injury. We specify in vivo that the miR-146a mimic could relieve myocardial I/R injury after 45 min of ischaemia and 1 week of reperfusion, reduce the infarction area and fibrosis area, and improve cardiac function.

During IR, ROS are mainly produced by the mitochondrial respiratory chain and NOx family enzymes. Other sources of ROS include xanthine oxidase (XO) and uncoupled nitric oxide synthase (NOS) [19]. To date, seven NOx subtypes (NOX1-5 and duox1-2) have been identified, among which NOX2 and NOX4 are the most abundantly expressed in the heart [20]. NOX2- and NOX4-mediated oxidative damage is very important during reperfusion. Oxygen supply during reperfusion provides a substrate for NOX-mediated ROS production [17]. Cardiac-specific NOX4-knockout mice showed decreased ROS and infarct size after IR, suggesting that NOX4 in cardiomyocytes plays a major role in mediating IR injury [21]. Surprisingly, mice with both NOX2 and NOX4 double knockout exhibited exacerbated myocardial injury [21]. A previous study reported that a miR-146a-5p mimic increased ROS production in SH-SY5Y cells [22]. However, An et al. reported that overexpression of miR-146a reduced ROS generation in H9c2 cardiomyocytes by increasing ErbB4 [18]. This paradox may account for different cell types as well as different miR-146 induction. In this study, we found that the miR-146a mimic decreased H/R- and I/R-induced ROS generation in cardiomyocytes and mouse hearts. Moreover, the miR-146a mimic reduced the level of MDA and diminished NADPH oxidase activity. Furthermore, we found that miR-146 directly inhibited the transcription level of NOX4 in cardiomyocytes. Recently, Wang et al. found that miRNA-146a inhibits NOX4 transcription in endothelial cells [23]. miR-146a was also reported to regulate NOX4-ROS in the human kidney cell line HK-2 [24]. In our study, we found that miR-146a could target the promoter region of NOX4 in cardiomyocytes. The miR-146a inhibitor enhanced the transcription level of NOX4. NOX4 silencing abolished the effects of the miR-146a inhibitor. This suggests that miR-146a inhibits ROS generation by directly inhibiting the transcription of NOX4, thus exerting cardioprotective effects during myocardial IR injury. However, the baseline expression of NOX4 was not changed after miR-146a mimic treatment, and other factors may regulate the transcription and translation of NOX4 under physiological conditions.

We also observed a reduction in inflammation in miR-146a mimic-treated mice and cardiomyocytes. During the myocardial infarction phase, acute inflammation leads to endothelial dysfunction and left ventricular pathological remodelling [25]. During the reperfusion phase, ROS can directly mediate the recruitment of neutrophils to the vascular inflammatory region and increase the inflammatory cascade by releasing various cytokines, leading to the activation of resident immune cells in heart tissue [26]. The anti-inflammatory effect of the miR-146a mimic may be a secondary result of anti-ROS generation. Reduced ROS could decrease the activation of immune cells and reduce cardiomyocyte injury. We observed that the number of CD68-labelled macrophages was reduced after miR-146a mimic injection. The inflammatory response in cardiomyocytes itself was also inhibited by the miR-146a mimic. Another anti-inflammatory effect of miR-146a may occur via P38 inhibition. P38, as a family of MAPK cascades, responds to stresses, such as oxidant stress, osmotic shock, infection, and cytokine exposure. P38 is rapidly activated within a few minutes of exposure to ROS, and P38 inhibition is reported to be sufficient to achieve anti-inflammatory effects in the heart [27]. We observed reduced P38 phosphorylation in miR-146a mimic-treated cardiomyocytes and mouse hearts. P38 injection may be a second effect of miR-146a on NOX4. The reduced P38 activation in the miR-146a mimic may be mediated by ROS inhibition, subsequently leading to inflammation inhibition in the heart.

Taken together, these results show that the miR-146a mimic protects against myocardial IR injury via the NOX4-ROS pathway, and the miR-146a mimic may become a promising treatment approach for patients with AMI who are subjected to coronary revascularization.

## Data Availability

Data are available on request.
